# Dielectric Elastomer Actuator Driven Soft Robotic Structures With Bioinspired Skeletal and Muscular Reinforcement

**DOI:** 10.3389/frobt.2020.510757

**Published:** 2020-12-15

**Authors:** M. Franke, A. Ehrenhofer, S. Lahiri, E.-F. M. Henke, T. Wallmersperger, A. Richter

**Affiliations:** ^1^Institute of Semiconductors and Microsystems (IHM), Technische Universität Dresden, Dresden, Germany; ^2^Institute of Solid Mechanics, Technische Universität Dresden, Dresden, Germany; ^3^PowerOn Ltd., Auckland, New Zealand

**Keywords:** dielectric elastomer actuator, soft robot, modelling and simulation, bimorph actuation, anisotropy, skeleton, silicone, pre-stretch

## Abstract

Natural motion types found in skeletal and muscular systems of vertebrate animals inspire researchers to transfer this ability into engineered motion, which is highly desired in robotic systems. Dielectric elastomer actuators (DEAs) have shown promising capabilities as artificial muscles for driving such structures, as they are soft, lightweight, and can generate large strokes. For maximum performance, dielectric elastomer membranes need to be sufficiently pre-stretched. This fact is challenging, because it is difficult to integrate pre-stretched membranes into entirely soft systems, since the stored strain energy can significantly deform soft elements. Here, we present a soft robotic structure, possessing a bioinspired skeleton integrated into a soft body element, driven by an antagonistic pair of DEA artificial muscles, that enable the robot bending. In its equilibrium state, the setup maintains optimum isotropic pre-stretch. The robot itself has a length of 60 mm and is based on a flexible silicone body, possessing embedded transverse 3D printed struts. These rigid bone-like elements lead to an anisotropic bending stiffness, which only allows bending in one plane while maintaining the DEA's necessary pre-stretch in the other planes. The bones, therefore, define the degrees of freedom and stabilize the system. The DEAs are manufactured by aerosol deposition of a carbon-silicone-composite ink onto a stretchable membrane that is heat cured. Afterwards, the actuators are bonded to the top and bottom of the silicone body. The robotic structure shows large and defined bimorph bending curvature and operates in static as well as dynamic motion. Our experiments describe the influence of membrane pre-stretch and varied stiffness of the silicone body on the static and dynamic bending displacement, resonance frequencies and blocking forces. We also present an analytical model based on the Classical Laminate Theory for the identification of the main influencing parameters. Due to the simple design and processing, our new concept of a bioinspired DEA based robotic structure, with skeletal and muscular reinforcement, offers a wide range of robotic application.

## Introduction

Biological evolution has revealed versatile motion types and structures culminating in the skeletal and muscular systems of vertebrate animals (Marshall, 1980). The effective, stable, and powerful interactions between skeleton, antagonistic muscles, ribbons, and joints have inspired researchers to transfer that ability into technical motion for artificial muscles or actuators in robotic systems (Bar-Cohen, 2016). However, replacing muscles and tissues by technical polymer materials is challenging, especially providing large strokes and generating high force output, while creating entirely soft devices. Furthermore, a smart integrated actuation system is needed, which typically consumes a significant amount of energy or consists of hard materials, as can be seen in the hydraulically driven autonomous soft robotic fish for 3D swimming (Katzschmann et al., 2016). Also fully soft actuator systems have been developed, e.g., a microfluidically and pneumatically driven autonomous working Octobot (Wehner et al., 2016) or a pneumatically driven multigait soft robot (Shepherd et al., 2011). These systems lack a stabilizing skeleton reinforcement and suffer from an obvious low movement speed. In the case of a given Octobot construction, the movement is inherent at a certain level of chemical concentration and cannot immediately be changed as the catalytical chemical decomposition of hydrogen peroxide produces a defined gaseous volume and leads to the tentacle movement. Furthermore, the reaction is quite sensitive to temperature changes and can decrease the precision of the displacement. The interaction and control of the system which commonly uses electrical signals is complicated. Also, in oscillatory operation, thermal influences on actuation and breakdown of DEAs have to be considered (Kleo et al., 2020).

Therefore, a direct electrical control of robotic actuation is preferred and can be achieved by dielectric elastomers (DE), which already have shown promising capabilities as artificial muscles for driving versatile biomimetic structures (Gu et al., 2017). For instance, DE are applied in a bimorph actuator (Goh and Lau, 2010), a fishtail (Berlinger et al., 2018), a transparent swimming soft robot (Christianson et al., 2018) or a caterpillar (Henke et al., 2017). They can simultaneously act as dielectric elastomer actuators generating large strokes and as sensors monitoring themselves (Henke et al., 2017). However, to generate significant amounts of stroke and force for actuation, membrane type actuators have to be pre-stretched to operate at their optimum work point (Kofod, 2008). Further advantages of pre-stretching the DE membrane are removing of electromechanical instabilities and enhancing breakdown field strength (Gu et al., 2017). However, it is difficult to integrate pre-stretched membranes in entirely soft robotic structures as instantaneous deformation occurs (Rosset et al., 2014; Araromi et al., 2015).

In this contribution, we present a simple, novel way to integrate DEAs, made of pre-stretched membranes, into soft robotic structures without any undesired bending. The robot possesses a bioinspired skeleton, integrated in a soft silicone body, for stabilizing the structural integrity and defining the degrees of freedom. This movement is stimulated by two antagonistic DEA artificial muscles made of isotropic pre-stretched membranes that are bonded to the body element. This allows a bimorph-bending curvature in the desired direction. The arrangement of this DEA pair allows a precise displacement in static mode and large and fast displacements in dynamic mode, due to resonance effects. In this study we investigated the influence of the pre-stretch value of the DE membranes and the stiffness of the silicone body material on the static and dynamic displacement of a bimorph-type bioinspired robot. Furthermore, the applicable blocking force of the robotic structure and the achievable maximum velocity and acceleration are estimated.

## Mechanism

### DEA Operation Principle

**Figure 1:**
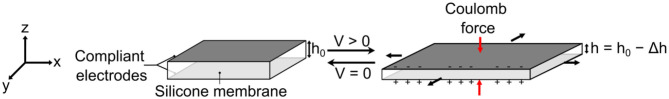
Working principle of a DEA, showing a voltage induced charging of the electrodes of the DEA capacitor structure. Coulomb forces lead to an attraction of the compliant electrodes and reduction of the initial film thickness **h**_**0**_ and an aerial expansion simultaneously.

DEAs possess a soft plate capacitor structure consisting of a flexible elastomer membrane, usually made of silicone or acrylic membranes, which are sandwiched between two compliant, conductive electrodes (see Figure 1) (Pelrine et al., 1998). To achieve reliable electrode flexibility, many different concepts and fabrication processes have been developed, wherein carbon-based materials like pure carbon powder, carbon grease or carbon-silicone-composites are most commonly used (Rosset and Shea, 2013). Application of high voltages, typically within the range of 1–5 kV, charges the compliant electrodes and generates a compression of the DEA membrane with an initial film thickness *h*_0_ due to the Coulomb forces. Due to its incompressibility, the polymer membrane gets compressed in *z*-direction with a certain thickness decrease *h* and expands in its lateral dimensions simultaneously. The elastic behavior of the DEAs causes a reversible reconfiguration of the initial shape and initial film thickness after discharging the electrodes (Kofod, 2008).

To achieve maximum performance, DEAs have to be pre-stretched (Kofod, 2008). Please note that the term λ^pre^ is used to denote the initial tensile deformation of the DEA, before it is added to the backbone structure. The same effect can also be called pre-strain with the according definition of technical strain ε^pre^ = λ^pre^ − 1. The pre-stretch of the actuators plays a significant role in the overall behavior of the DEA structure. This has several reasons: pre-stretching of the membrane reduces its thickness and, thus, scales the Maxwell stress, and the compression force. This effect scales quadratically with the DEA thickness. From the mechanical point of view, the role of buckling instability has to be considered as well: In active-passive composite setups for actuation purposes—e.g., when the passive material is used as a stiffening backbone—the active material is mostly in a constrained setup (Ehrenhofer and Wallmersperger, 2020). In such setups, an elongation of a thin structure, i.e., a structure with one or more dimensions much smaller than the primary direction(s), leads to buckling instabilities (Biot, 1963). Pre-tension of a DEA strip—in the current setup realized by pre-stretching the membrane—circumnavigates this problem. However, the pre-stretch must be accordingly high so that throughout the actuation, the applied stress is always lower than the pre-stress, or buckling will occur.

### Design and Setup

**Figure 2:**
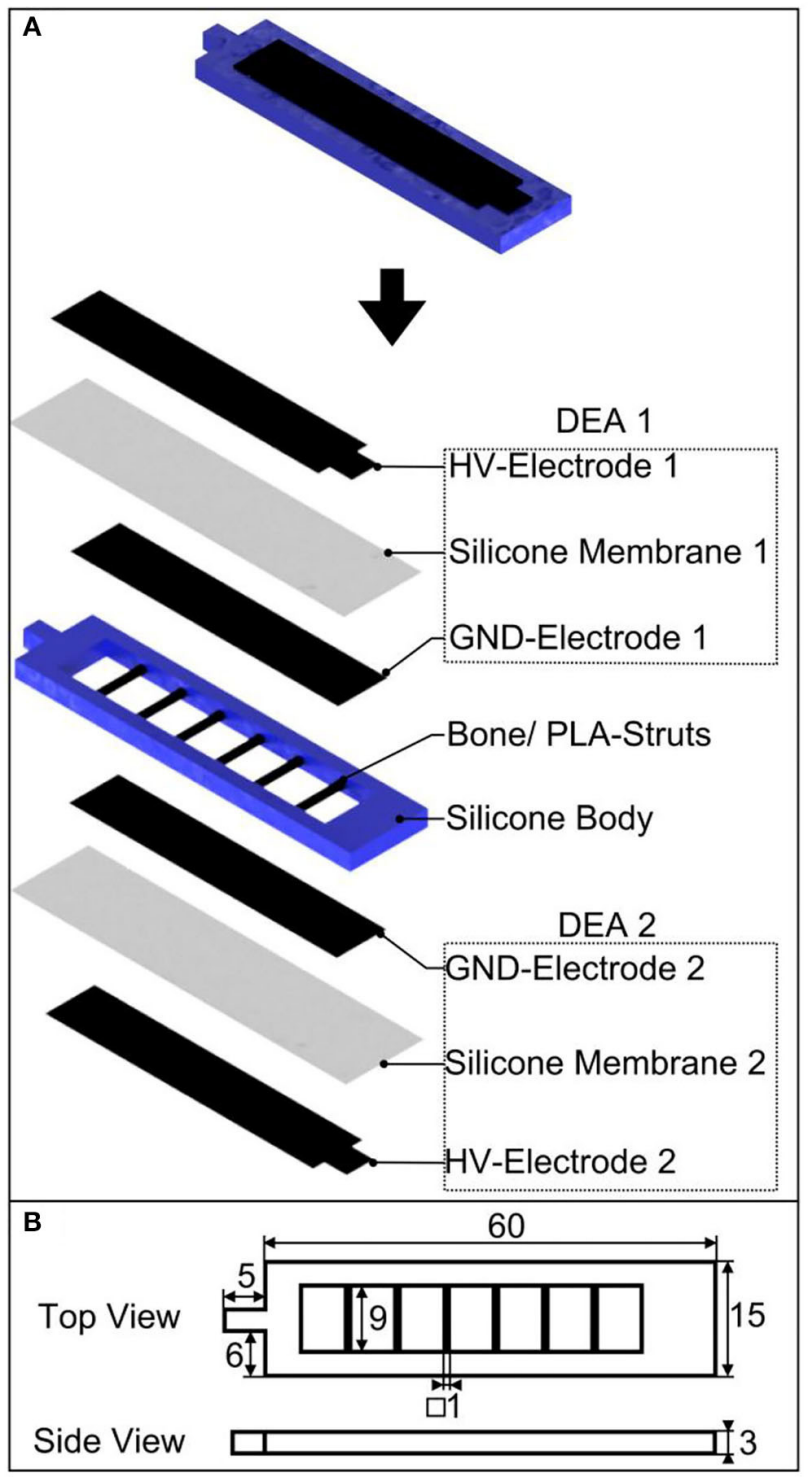
Bioinspired soft robotic structure with an antagonistically working DEA artificial muscle pair and integrated bones for anisotropic bending stiffness; **(A)** design and setup as exploded representation, **(B)** geometric dimensions in mm.

The soft robotic structure presented in the current work, comprises a multilayer setup, which is schematically shown in Figure 2A. The setup consists of two DEA artificial muscles on the top and bottom of a soft silicone body. Both DEA membranes possess the same defined equibiaxial pre-stretch (λ_pre_), covered with compliant electrodes, where the outer electrode represents the high voltage (HV) electrode and the inner one the ground (GND) electrode. The GND electrodes are fixed toward the central body element and connected to the same ground potential. The silicone body itself is flexible, with integrated stiff PLA struts, allowing anisotropic bending stiffness of the entire soft robotic structure, resulting in high flexibility in the longitudinal direction and a high bending stiffness in the transverse direction simultaneously. Figure 2B depicts the geometric dimensions of the overall structure.

### Bending Mechanism and Analytical Modeling

**Figure 3:**
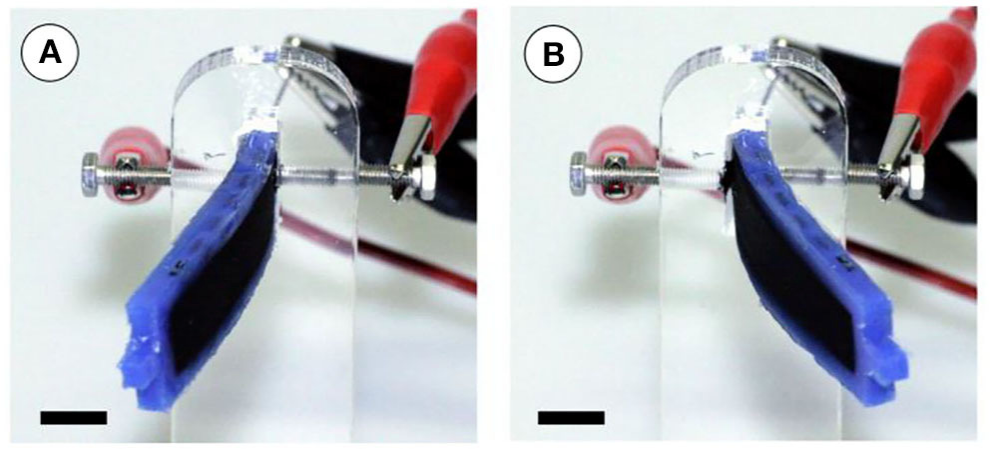
Working principle of the soft robotic structure with two antagonistically working DEA muscles during dynamic testing. In **(A)** the right DEA is activated and the robot bends left. In **(B)** the left DEA is activated and the robot bends right (scale bar is 10 mm).

Figure 3 depicts the deflection of the structure during testing, to illustrate the working principle of the soft robotic structure. Both DEA artificial muscles cover the silicone body and possess the same defined pre-stretch. In the equilibrium state, the bending moments, which are generated by both DEA muscles and applied to the silicone body, are balanced and the structure stays straight. When the DEAs operate antagonistically, bending of the structure occurs in the opposite direction of the activated DEA. The reason for this behavior is the superposition of the (tensile) pre-stretch with the actuation strain that follows through lateral contraction in the activated membrane. This results in less force and, thus, less bending moment applied to the silicone body by the activated DEA. Depending on which DEA is activated, the robotic structure bends reversibly to the left or right direction. This allows a static or dynamic bending as can be seen in Supplementary Video SM1. A better view and explanation of the bending behavior is given by Figure 3A, in which the right DEA is activated and the robot is displaced to the left direction. Instead in Figure 3B, the left actuator is activated and the robot is displaced to the right.

The description of the layered system, for design purposes, can be achieved through different approaches. In the current work, we focus on (i) the analytical description using the two-dimensional Classical Laminate Theory (Reddy, 2003) and (ii) the full three-dimensional description. Both approaches have in common that the actuation of the DEA layers is realized by using the Temperature-Expansion-Model (Ehrenhofer et al., 2016, 2018).

In the current modeling approach, the actuation strain of the DEA is implemented using the analogy to thermal expansion, i.e., the area strain ε_*xx*_ = ε_*yy*_ = ε_in−plane_ is modeled with thermal expansion strain ε^therm^ and realizes following relation:

(1) $$\begin{array}{l} \varepsilon_{\text{in}-\text{plane}}\;\hat{=}\;\varepsilon^{\text{therm}}=\;\alpha \;\Theta . \end{array}$$

Here, α is a (pseudo-)thermal expansion coefficient and Θ a stimulus-difference (normalized to a reference value). Thus, the elongation of the plate-like (i.e., *l*_*x*_, *l*_*y*_ ≫ *l*_*z*_) DEA in *x-y*-plane due to the compression in *z*-direction, caused by the electrostatic force, is replicated. The adequate definition of ε^therm^ can be implemented in the context of the 3D continuum mechanical formulation as well as in simplified 2D and 1D theories.

Note that in the derivation of 2D theories, like plate or shell descriptions, the *z*-direction is assumed as the neutral direction. In combination with the thinness-criterion, this leads to the assumption of plane stress state (Reddy, 2003). Since the main criteria of these theories are violated in DEA structures, 2D theories must be applied with extreme caution. This is also valid for the use of 1D theories like Bernoulli beam theory, in which a one-dimensional stress state is assumed. Layered composite shell structures require more complex assumptions, which makes the analytical solution process more difficult, even calling for specialized discretization methods (Kulikov and Carrera, 2008). In those cases, a full three-dimensional modeling approach, solved by using the Finite-Element-Method, might be more efficient.

Note that the applied material model for the analytical description with CLT is linear. The underlying assumption is that for composite setups designed for bending purposes, small strains can lead to large strokes. This is due to the effect of active-passive stiffness pairing, i.e., both material and geometrical parameters play a role in the levering effect (Ehrenhofer and Wallmersperger, 2020). This is also known from simple bimetal strips where very small temperature changes and small thermal expansion coefficients lead to large tip displacement, nevertheless. From the viewpoint of modeling, a Young's modulus (elastic modulus) is gained through a linearization at a working point, e.g., the pre-stretched DEA.

### Description Using the Classical Laminate Theory

The Classical Laminate Theory is a common approach for multilayer composite systems (Reddy, 2003). It is based on the description of every lamina as a homogenized structure with possible direction dependent properties. It is valid for a certain set of prerequisites: small deformations, linear elasticity, constant layer thickness, Bernoulli hypothesis and ideal connection of the layers. The derivation of the expression for the curvature is shown in the Modeling Appendix. The curvature of the bending of the composite is derived there.

**Figure 4:**
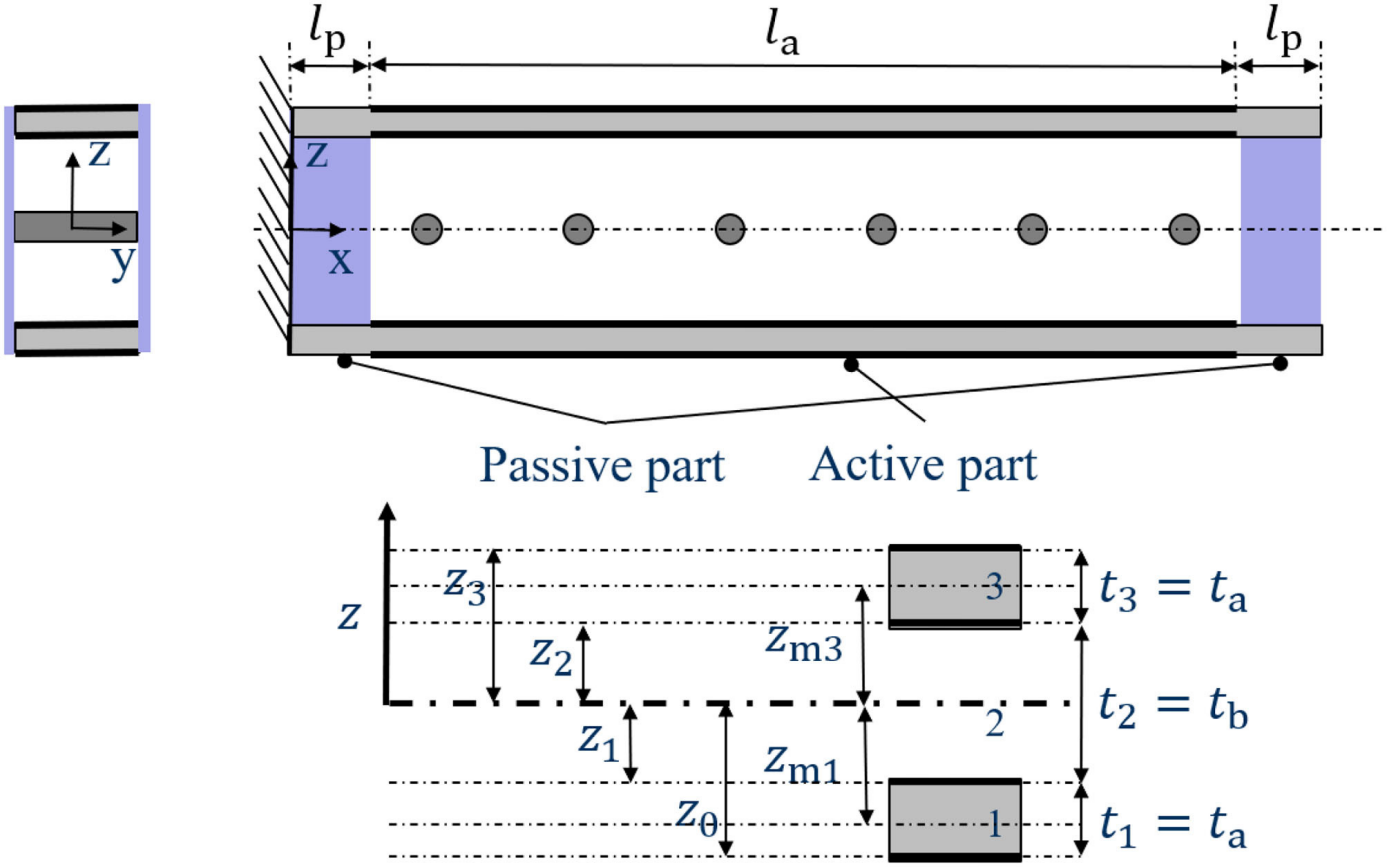
Side-view of the cut through the cantilever-like structure and according coordinate definition for the Classical Laminate Theory.

The actual 1D setup in *x*-direction (side-view according to Figure 4) is needed to derive the bending line and the respective tip displacement for comparison to the experimental values. The bending line is separated into three parts, whereas the only active part is in the middle.

The tip displacement, i.e., the displacement of the right side when the composite system is fixed on the left side, is derived as

(2) $$\begin{array}{l} w_{\text{Tip}}=w\left(x=l_{a}+2\;l_{p}\right)\\ \;=\frac{{3\;l}_{a}\;E_{a}\;\left(t_{a}+t_{b}\right)\left(\alpha_{1}\Theta_{1}-\alpha_{3}\Theta_{3}\right)\;\left(l_{a}+2\;l_{p}\right)}{8\;E_{a}t_{a}^{3}+24\;E_{a}\;t_{a}^{2}\;t_{p}+24\;E_{a}\;t_{a}t_{b}^{2}+E_{b}t_{b}^{3}}. \end{array}$$

The derivation of Equation (2) can be found in the Modeling Appendix (Supplementary Material 1). Here, for *i* = *a, p* with active (a) and passive (p), the material and geometry parameters are the lengths *l*_*i*_, layer thicknesses *t*_*i*_ and Young's moduli *E*_*i*_. The expansion strains α_*i*_ Θ_*i*_ for the active layers 1 and 3 include pre-strain and actuation. Equation (2) is only valid for small deformations, i.e., a total tip displacement of generally less than half the beam height. The scope of the analytical approach is therefore very narrow; however, it can be used to identify the crucial parameters in the system. For the description of the complete bending process, a three-dimensional model is needed.

The developed model, based on the CLT, is now applied with the experimental values. As can be seen in the Equation (2), the pre-strains in the DEAs (layer 1 and 3) cancel out in $\alpha_{1}\Theta_{1}-\alpha_{3}\Theta_{3}=\left(\varepsilon_{1}^{\text{pre}}+\varepsilon_{1}^{\text{actuation}}\right)-\left(\varepsilon_{3}^{\text{pre}}+\varepsilon_{3}^{\text{actuation}}\right)$ and only the difference between the actuation strains remains. However, the pre-strain still plays a role in the layer thickness. The actuation strain in height direction is

(3) $$\begin{array}{l} \varepsilon_{zz}^{\text{actuation}}\left(V\right)=-\epsilon_{0}\epsilon_{r}\frac{V^{2}}{E_{\text{ELASTOSIL}}\;t_{\text{ELASTOSIL},\text{ pre}-\text{stretched}}^{2}} \end{array}$$

where $\epsilon_{0}=8.854\cdot 10^{-12}\frac{\text{As}}{\text{Vm}}$ is the vacuum permittivity, ϵ_*r*_ = 2.8 the relative permittivity between the electrodes, and *E*_ELASTOSIL_ the Young's modulus of the ELASTOSIL membrane (experimental values are shown in section DEA). There is no stiffening effect of the electrodes due to the described fabrication process: The pre-strain is only applied to the ELASTOSIL membrane, then the electrodes are added. In Equation (3), the thickness of the ELASTOSIL membrane *t*_ELASTOSIL, pre−stretched_ depends on the multiaxial in-plane pre-stretch $\lambda_{x}=\lambda_{y}=\lambda^{\text{pre}}$ via volume constancy (λ_*x*_λ_*y*_λ_*z*_ = 1)

(4) $$\begin{array}{l} t_{\text{ELASTOSIL},\text{ pre}-\text{stretched}}=t_{\text{ELASTOSIL},0}\frac{1}{{\left(\lambda^{\text{pre}}\right)}^{2}} \end{array}$$

with the initial membrane thickness of *t*_ELASTOSIL, 0_ = 100 μ*m*. Thus, the actuation strain $\varepsilon_{zz}^{\text{actuation}}\left(V\right)$, which depends on the voltage *V* = 0…5 kV can be calculated.

The actuation strain in *z*-direction is now transformed to in-plane strain. Please note that the actuation strain equally contributes to strains in *x*- and *y*-direction, therefore λ_*x*, act_ = λ_*y*, act_ = λ_in−plane, act_, which leads to

(5) $$\begin{array}{l} \lambda_{x,\text{act}}\lambda_{y,\text{act}}\lambda_{z,\text{act}}=\lambda_{\text{in}-\text{plane},\text{act}}^{2}\lambda_{z,\text{act}}=1 \end{array}$$

(6) $$\begin{array}{l} \varepsilon_{3}^{\text{actuation}}=\varepsilon_{xx}^{\text{actuation}}=\lambda_{\text{in}-\text{plane},\text{act}}-1=\frac{1}{\sqrt{\lambda_{z,\text{act}}}}-1\\ \;=\frac{1}{\sqrt{\varepsilon_{zz}^{\text{actuation}}+1}}-1 \end{array}$$

With the described simulation parametrization, the following static tip displacement of the robot according to Equation (2) can be calculated, and is compared with the experimental data, see Figure 8. Here, *E*_*a*_ = *E*_ELASTOSIL_ because, as explained above, the electrodes do not play a role in the pre-strain and actuation strain. For the Young's modulus of the passive material *E*_*p*_, different experimental values for the different materials are inserted, see section Investigation of the Materials Young's Moduli.

## Fabrication

### DEA

**Figure 5:**
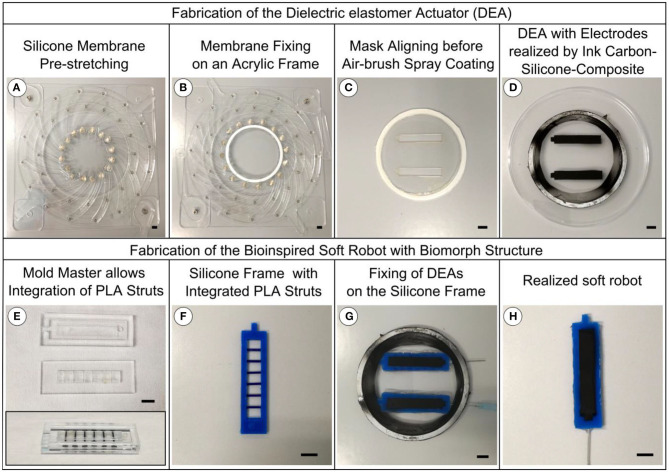
Fabrication process of the DEA and the bioinspired soft robotic structure. **(A)** Pre-stretching of the silicone membrane. **(B)** Fixing the silicone membrane to an acrylic frame using an adhesive RTV silicone. **(C)** Shadow masks for processing the compliant electrodes by aerosol deposition, using an air-brush, on the top and bottom side of the silicone membranes. **(D)** Finalized DEA membrane. **(E)** Acrylic mold, including the bone elements, for injection molding the silicone body. **(F)** Silicone body with integrated bone elements. **(G)** Bonding of the silicone body on top of the previously prepared DEA membrane, still fixed on an acrylic frame, by an adhesive RTV silicone. Afterwards, a second DEA membrane is bonded on top of the body. **(H)** Finalized soft robotic structure. Scale bar is 10 mm.

The fabrication process of the DEAs is described in Figures 5A–D. In order to prepare the DEAs, commercially available 100 μm thick silicone elastomer membranes (ELASTOSIL 2030 by Wacker Chemie AG, Germany) are fixed in an in-house made pre-stretching device using magnetic clamping (Figure 5A) and pre-stretched to the desired equibiaxial values of λ_pre_ = 1.1, 1.3, 1.5, and 1.7, respectively. After pre-stretching, the silicone membrane is fixed to an acrylic frame using an adhesive RTV silicone (RS PRO Silicone) (Figure 5B). The compliant electrodes are fabricated by aerosol deposition, using an air-brush and shadow masks (Figure 5C), on the top and bottom side of the silicone membranes. The ink is produced by mixing carbon black powder (Vulcan XC72, Cabot, CA, USA) with a two-component silicone elastomer rubber (Ecoflex 00-10 by Kaupo, Germany). To adjust the viscosity, Heptane is used as a solvent (Ashby et al., 2019). After applying the ink, the electrodes are cured at 40°C for 12 h. Figure 5D shows the finalized DEA membrane.

### Soft Robotic Structure

The fabrication process of the soft bimorph structure is shown in Figures 5E–H. The silicone body is injection molded in an acrylic mold, including the bone elements, consisting of 3D printed PLA struts (Figure 5E). In order to study the influence of different Young's moduli on the static and dynamic behavior of the entire system, three different silicones, with different Young's moduli are used: Ecoflex 00-10 (Kaupo, Germany), Moldstar 30 (Kaupo, Germany), and Sylgard 184 (Dow Corning, USA). The two-component silicone materials are processed consecutively by sufficient A:B-mixing (A:B = 1:1 Ecoflex 00-10 and Moldstar 30, A:B = 10:1 Sylgard 184), degassing, injection into the mold, and finally heat curing at 60°C for 12 h. The resulting silicone body with integrated bone elements (Figure 5F) is firstly bonded on top of the previously prepared DEA membranes, still fixed on an acrylic frame, by the adhesive RTV silicone (Figure 5G). Afterwards, a second DEA membrane is bonded on top of the structure by the same process. The GND contacts are realized by a cannula that is pierced through the silicone body. After curing the RTV silicone, the bodies are cut out of the acrylic frames. Figure 5H depicts a final structure.

### High Voltage Supply

The high voltage signals, driving the two DEAs 180° phase-shifted, are supplied by the open-source low-current high voltage power supply: Peta-pico-Voltron, previously presented by Schlatter et al. (2018). The high voltage power supply has three separately working channels able to provide 5 kV, either continuous, or as square waves with a frequency range between 1 mHz and 1 kHz. The channels can be run simultaneously and phase-shifted.

## Experiments

### Investigation of the Materials Young's Moduli

To investigate the Young's modulus of the DEA membrane material ELASTOSIL 2030 and the silicone support structure materials Ecoflex 00-10, Moldstar 30 and Sylgard 184 tensile testing experiments are conducted. Ecoflex 00-10, Moldstar 30, and Sylgard 184 are processed as reported in section DEA as 200 μm films by the automatic film applicator ZAA 2300 (Zehnter, Switzerland). ELASTOSIL 2030 membrane with 100 μm thickness is used as received. The membranes were cut into samples according to DIN 53504 (5 per material) and tensile testing is performed by the testing machine Z005/TN (ZwickRoell GmbH & Co.KG, Germany). Stress/strain curves are used to calculate the Young's moduli *E*_*i*_ of each material by linear fitting up to a strain of 10% (see Table 1).

**Table 1:** Young's moduli of the applied silicone materials.

**Material**	***E*_*i*_ in *kPa***
ELASTOSIL 2030	1400.4 ± 33.4
Ecoflex 00-10	36.53 ± 1.45
Moldstar 30	805 ± 17.1
Sylgard 184	1301.1 ± 27.5

### Recording of the Displacement (Static/Dynamic), Velocity, and Acceleration

**Figure 6:**
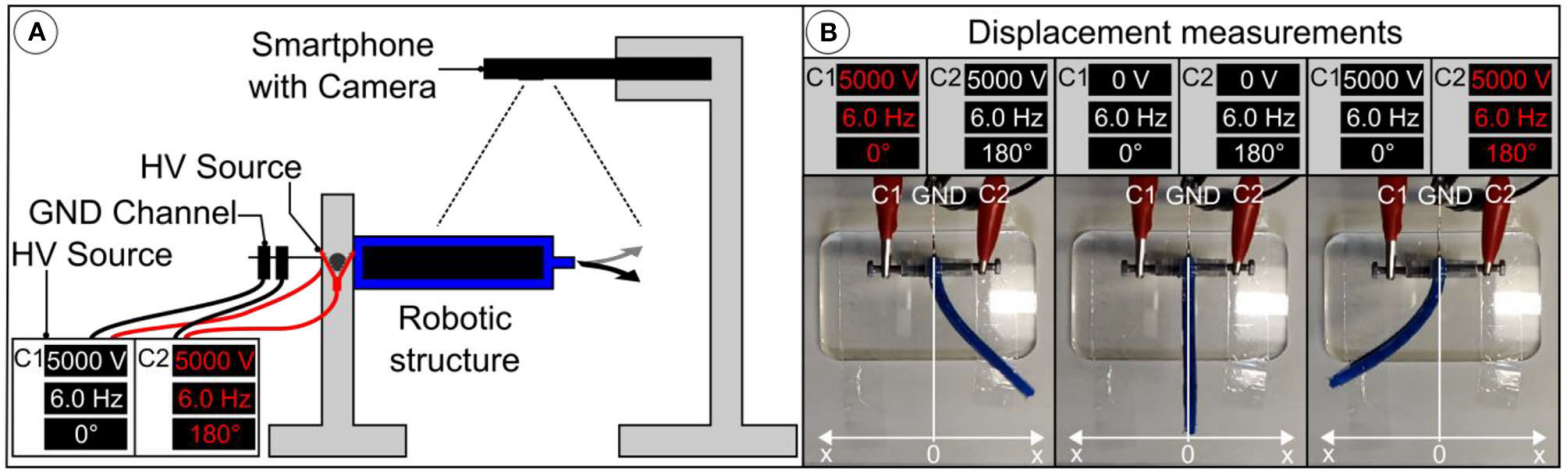
Measurement of the displacement while running in static or dynamic mode. **(A)** Scheme of the experimental setup. **(B)** Video frames showing the left and right displacement x at maximum in dynamic mode depending on the voltage and frequency. Note that both actuators of the bimorph robotic structure are working 180° phase shifted while the activated one shows the red colored channel C1 or C2.

To investigate the influence of membrane pre-stretch and mechanical stiffness of the silicone material, used for the body, on the bending displacement (static), bending amplitude and frequency behavior (dynamic) of the robotic structure, a series of experiments, measuring the bending displacement for a voltage range up to 5 kV are conducted. Velocity and acceleration are measured during dynamic experiments. The experimental setup is shown in Figure 6A. The actuator is attached to a mount and the electrodes are connected to two channels of the high voltage power supply. A smartphone camera with a high-speed sensor is mounted vertically above the setup to capture videos of the actuator at a speed of 480 fps and a resolution of 720p. Figure 6B shows three captured video frames of the robotic structure at distinct displacements, depending on the voltage and frequency of the square waves. The driving voltage of the actuator is increased from 3,000 to 5,000 V in steps of 500 V using two channels of the power supply. For each voltage, a frequency, ranging from 2 Hz to 10 Hz, is applied. A step of 1 Hz is used for frequencies from 2 Hz to 6 Hz and from 8 Hz to 10 Hz. A step of 0.2 Hz is used for the frequencies from 6 Hz to 8 Hz to observe the resonance frequency carefully. A 180° phase-shift is maintained between the two channels, for maximum actuation. For each of the applied frequencies, a video of ~5 s duration is captured. This procedure is repeated for robots with the body material Moldstar 30 and DEAs made from membranes having a pre-stretch of λ_pre_ = 1.1, 1.3, 1.5, and 1.7. The same process is repeated for DEAs with membranes stretched to λ_pre_ = 1.5 but different body materials (Sylgard 184 and Ecoflex 00-10). The Video SM2 shows the dynamic displacement of a robotic structure from the camera's perspective as a reference.

### MATLAB Tool for Video Analysis

**Figure 7:**
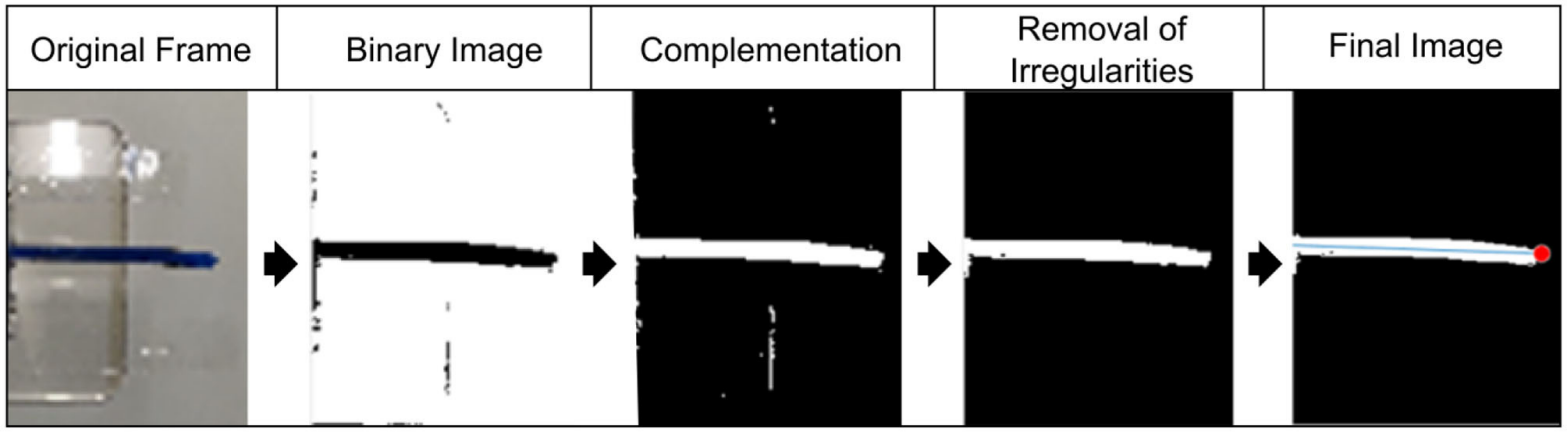
Video analysis with processing steps to realize binary images for automated displacement, velocity, and acceleration measurements. The robot size was used to calibrate distance measurements.

The video data is extracted (in the form of text files) from the captured videos using a MATLAB GUI tool for all experiments. 300 frames of each video are processed to extract necessary data. The steps of processing for each frame are shown in Figure 7. At first, a frame of the original video is converted into a binary image and its complement. After removing irregularities, the final image is received. The robot size is used to calibrate distance measurements. Furthermore, the position of the tip of the robotic structure is found in pixels and written to the text file. The amplitude is calculated in pixels and then converted to distance in millimeters with the appropriate correction factor. This finally allows the displacement, the velocity, and the acceleration of the robotic structures to be derived. The velocity is measured at the zero position and the acceleration at the largest displacement of the robotic structure.

### Blocking Force

The blocking forces, which the soft robotic structure can apply during static displacement, is measured using a force sensor (F_Max_ = 2 N, type 8432-5002-V0F00000, by burster präzisionsmesstechnik gmbh & co kg, Germany). The sensor is moved precisely to the tip of the robotic structure, using a *x-y* micrometer stage, until a minimum contact force is detected. The blocking force measurements is conducted for all samples and conditions investigated during static measurements described in section Recording of the Displacement (static/dynamic), Velocity, and Acceleration.

## Results and Discussion

### Static Displacement

**Figure 8:**
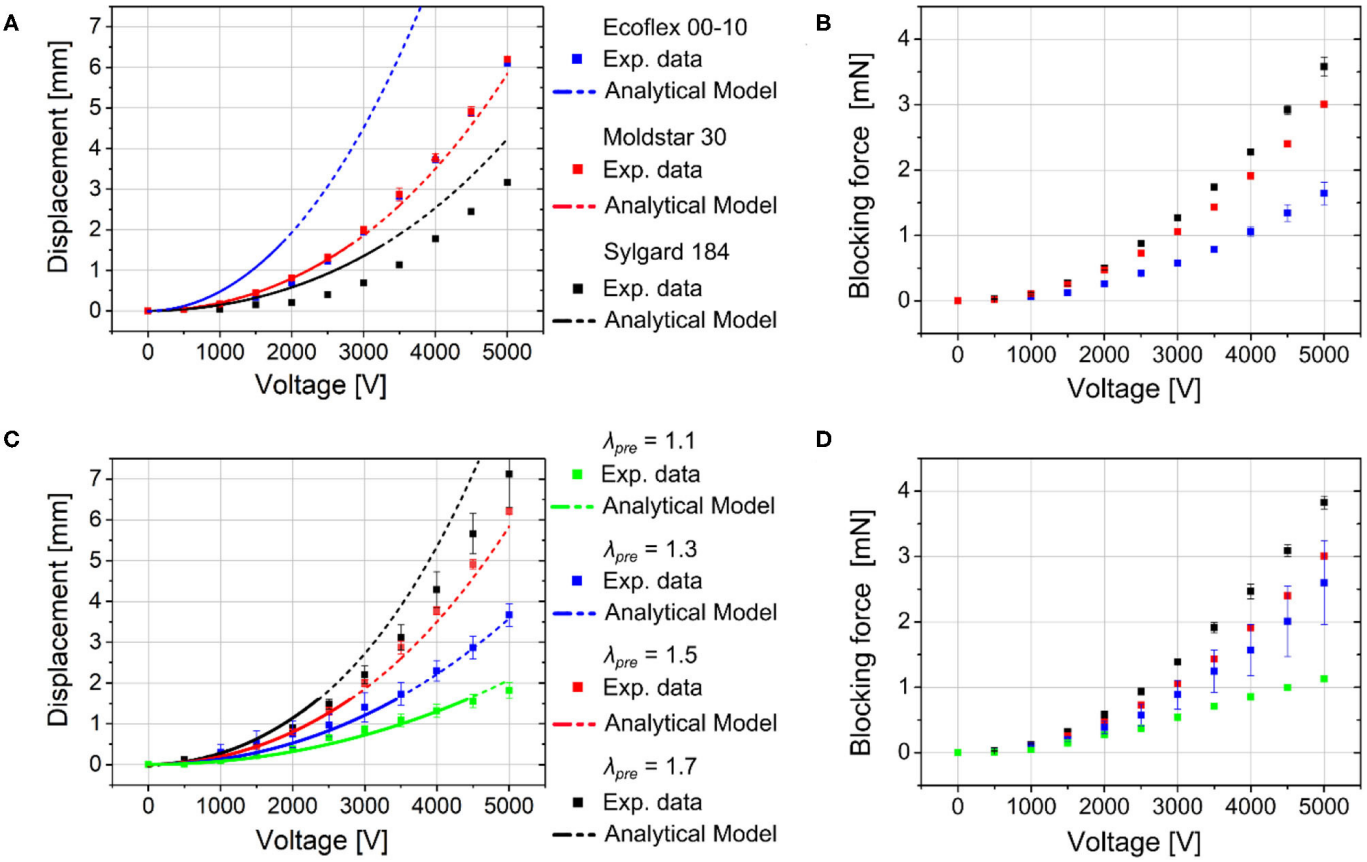
Static displacements (data from experiment and analytical modeling) and applied blocking forces of the robotic structures depending on the applied voltage with **(A,B)** different silicone body materials (Ecoflex 00-10, Moldstar 30, Sylgard 184) with constant pre-stretched DE membrane (λ_pre_ = 1.5) and **(C,D)** varied pre-stretch (λ_pre_ = 1.1, 1.3, 1.5, 1.7) of the DEA silicone membranes with Moldstar 30 as silicone body material.

Bending behavior of the soft robotic structure is investigated with regards to the mechanical influence of the silicone body under static actuation at different applied voltages. Figure 8A depicts the displacement of robotic structures made of the three body materials, Ecoflex 00-10, Moldstar 30, and Sylgard 184, which differ in mechanical stiffness and density. The DEA membranes are uniformly stretched to λ_pre_ = 1.5. The voltage vs. displacement curves show the typical quadratic behavior. As Sylgard 184 is stiffer than the other materials, the displacement 3.1 mm at 5 kV is only half of the displacement of the softer structures with 6.1–6.2 mm. Interestingly, the structure with the highly flexible Ecoflex 00-10 did not achieve higher displacement values. The reason is explained in the end of this section. Instead, increasing the stiffness of the body material enhances the blocking force of the robotic structure, as can be seen in Figure 8B.

Additionally to a variation of the silicone body material, we conduct investigation regarding different pre-stretched DEA membranes λ_pre_ = 1.1, 1.3, 1.5, 1.7 with uniformly chosen silicone body material Moldstar 30 and measured the robot's displacement (see Figure 8C). Here, a distinct increase of displacement from about 2 (λ_pre_ = 1.1)−7 mm (λ_pre_ = 1.7) at 5 kV can be observed when the membrane pre-stretch is increased. A higher pre-stretch caused a better electromechanical coupling since the membrane layer of the capacitor structure got thinner, which increased the resulting thickness strain ε_*zz*_ of the activated DE membrane, see Equation (3). This reduces the mechanical stress applied to the silicone body. Large differences of the mechanical stress values between the activated and non-activated DEA enhance the robot's displacement. Furthermore, hyperelastic materials like silicone elastomer ELASTOSIL 2030 show a minimum Young's modulus in stress-strain curves at a certain pre-stretch due to high non-linearity. This allows a higher displacement at a certain pre-stretch value. In Figure 8D, the pre-stretch change from λ_pre_ = 1.1–1.7 facilitates nearly four times higher blocking force up to 4 mN (λ_pre_ = 1.7) during actuation at 5 kV, which can be explained by a better electromechanical coupling and a stiffening of the silicone membrane.

Furthermore, we carried out analytical calculations for the robot bending with the three different silicone body materials and varied the applied pre-stretch, described in section Bending Mechanism and Analytical Modeling. The calculated displacement values nicely fit to the experimental values. Deviations may be a result of fabrication variation, the influence of the silicone glue that is not considered in the analytical model and variation in the actual material stiffnesses. The large difference between experimental and simulation results for the Ecoflex 00-10 silicone body material can be attributed to the very low Young's modulus of this material that results in an undefined collapsing of the pre-stretch valued structure. Compare the actuators with Ecoflex 00-10 and Sylgard 184 silicone bodies in Supplementary Figure 7. The respective post-buckling state cannot be captured by a linear model as proposed above. The required non-linear 3D model is part of our future works.

Due to the assumptions and linearization used in the analytical model, it is only valid for voltages up to ~2, 000 *V*, even though the similarity between analytical and experimental results seems acceptable up to 5, 000 *V*. Therefore, only displacement values up to 0.5 · *t*_*b*_ can be reasonably compared with the experimental results. This is denoted by the dashed line for the modeling results.

However, due to the acceptable agreement with the experimental results, in the model's validity range, the model can be used to design this kind of actuator, i.e., the influence of different parameters can be compared in order to enhance the actuator performance.

For further insights about the role of the parts of the structure, a full three-dimensional model must be derived, which will be part of our future works. There, also the non-linearity of kinematics and material can be captured.

### Dynamic Displacement

**Figure 9:**
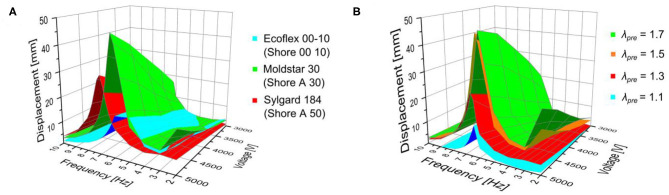
Results of the investigation concerning the dynamic displacement depending on the applied voltage and frequency of the robotic structures with **(A)** different silicone body materials (Ecoflex 00-10, Moldstar 30, Sylgard 184) with uniformly pre-stretched DE membrane (λ_pre_ = 1.50) and **(B)** varied pre-stretch (λ_pre_ = 1.1, 1.3, 1.5, 1.7) of the DEA silicone membranes with Moldstar 30 as silicone body material.

The investigations of the static actuation manifest a strong dependency of the robotic structure's actuation behavior on the membrane pre-stretch and silicone body material. Simultaneously, switching from static to dynamic (here: oscillatory) actuation mode is supposed to enhance the bending and displacement behavior as resonance effects multiply the achievable displacement values. Figure 9A depicts the dynamic displacement (here: amplitude) of the soft robotic structures with different body materials (Ecoflex 00-10, Moldstar 30, Sylgard 184) and uniformly chosen membrane pre-stretch λ_pre_ = 1.5. The dynamic displacements are shown in 3D charts and depending on the applied voltage and frequency, on which both DEAs operate 180° phase-shifted. The maximum displacements of all three actuator types are achieved at the 5 kV voltage maximum, but show distinct frequency maxima or resonance frequencies at 5 Hz and 17.2 mm (Ecoflex 00-10), 6.2 Hz and 46.6 mm (Moldstar 30) as well as 7 Hz and 31.2 mm (Sylgard 184). The resonance frequencies are arranged in a way which can be expected from silicone body materials with low to high stiffness. Herein, Moldstar 30 seems to be the optimal body material enabling high actuation with our system. Additional 2D charts in Supplementary Figures 3, 5, and Supplementary Figure 6 show the dynamic displacement of the three robots more precisely.

In addition to the variation of the different silicone body materials, we investigated the influence of membrane pre-stretch (λ_pre_ = 1.1, 1.3, 1.5, 1.7) on the dynamic displacement. Here, the silicone body material is Moldstar 30. In Figure 9B, the maximum displacements at 5 kV are increasing from 13.0 mm at 6.4 Hz (λ_pre_ = 1.1), 25.2 mm at 6.4 Hz (λ_pre_ = 1.3) to 46.6 mm at 6.2 Hz (λ_pre_ = 1.5), and stay nearly constant at 47.4 mm at 6 Hz (λ_pre_ = 1.7). Additional 2D charts in Supplementary Figures 1–4 show the dynamic displacement of the four robots more precisely.

As can be seen from the experimental values for the frequency-amplitude relation in Figure 9A, the resonance frequency changes for different materials. This can be attributed to (i) the change in Young's modulus, (ii) the different resulting length due to the coupling with the pre-stretched DEA and (iii) the different density of the materials. (i)–(iii) can be found in Equation (7). However, another effect that leads to a change in resonance frequency is a change in the dampening parameters, which will be part of our future investigations.

For different pre-stretches, only very minor variations can be found in Figure 9B. This is due to the fact that the effects (i) and (ii) above counteract: For higher pre-stretch, the Young's modulus gets smaller. At the same time, the length of the structure gets smaller, which influences ω_*r*_ in the 4th order. The quantification of this effect will be part of future studies.

The main influencing parameters to the resonance frequencies ω_*r,i*_ (for *i* the eigenmodes 1 and 2) can be estimated from the equation for linear oscillations

(7) $$\begin{array}{l} \omega_{r,i}=\;\kappa_{i}^{2}\sqrt{\frac{E_{x}I_{yy}}{mL^{4}}} \end{array}$$

where *E*_*x*_ is the Young's modulus in *x*-direction, *I*_*yy*_ the moment of inertia around the *y*-axis, *m* the mass and *L* the beam length.

Furthermore, we also measured the maximum velocity *v*_max_ and maximum acceleration *a*_max_ of the robotic structure. The data can be found in Supplementary Figures. The frequency depending behavior regarding the *v*_max_ and *a*_max_ values equals the dynamic displacement. By varying the DE membrane pre-stretch from λ_pre_ = 1.1–1.7 and using the same silicone body material Moldstar 30, both properties raise from *v*_max_ = 1.7–6.5 m/s and *a*_max_ = 11–38 m/s^2^, at an activation voltage of 5 kV. At the same time, the lowest values of *v*_max_ and *a*_max_ are obtained with the silicone body material Ecoflex 00-10 and a pre-stretch of λ_pre_ = 1.5.

## Conclusion

Artificial muscles based on DEAs have a great potential use in robotic systems, as they are soft, lightweight and are able to undergo large strokes. Here, we present a soft robotic structure, possessing a bioinspired skeleton, integrated into a soft body element, with an antagonistic working DEA artificial muscle pair. The finger-sized robotic structure (60 mm) showed an anisotropic, biomorphic bending behavior, due to stiff bone-like PLA struts, integrated into a silicone body element. During the experimental evaluation of the robot, a distinct biomorphic bending curvature is realized and can be analytically modeled using the Classical Laminate Theory. Depending on the optimized pre-stretch (λ_pre_ = 1.5–1.7) of the DEA membrane and stiffness of the silicone body material, fast and large dynamic displacements of about 47 mm are accomplished. In the same way, it was possible to optimize the blocking force from 1 to 4 mN. The investigations show how different parameters could improve the actuator performance of the robot for future application. In this way, it is reasonable to integrate our system in a real application like swimming robots with a fish tail.

## Data Availability Statement

The datasets generated for this study are available on request to the corresponding author.

## Author Contributions

MF and E-FMH conducted the concept, design, setup, and experiments of the project. AE and TW developed the analytical model of the bending mechanism. SL executed the experiments. AR provided advice about the fundamentals of this research topic. All authors composed through written, intellectual, or experimental contributions which approve of its publication, contributed to the article, and approved the submitted version.

## Conflict of Interest

E-FMH was employed by the company PowerON Ltd. The remaining authors declare that the research was conducted in the absence of any commercial or financial relationships that could be construed as a potential conflict of interest.

## Supplementary Material

Supplementary Video SM1 Performance of the dynamic bending behavior.

Supplementary Video SM2 Performance of the dynamic bending behavior during measurement shown in slow-motion.
