# Supplementary material for: Dielectric Elastomer Actuator Driven Soft Robotic Structures With Bioinspired Skeletal and Muscular Reinforcement
Source: Front Robot AI. 2020 Dec 15;7:510757. doi: 10.3389/frobt.2020.510757 (PMC7805688; doi:10.3389/frobt.2020.510757)
Supplement: Supplementary file 3 [file Data_Sheet_1.PDF]

Supporting Information

Dielectric elastomer actuator driven soft robotic structures with bioinspired skeletal and muscular enforcement

M. Franke*[1], A. Ehrenhofer[2], S. Lahiri[1], E.-F. M. Henke[1,3], T. Wallmersperger[2], A. Richter[1]

1 Chair of Microsystems, Institute of Semiconductors and Microsystems (IHM), Technische Universität Dresden, 01062 Dresden, Germany

2 Chair of Mechanics of Multifunctional Structures, Institute of Solid Mechanics, Technische Universität Dresden, 01062 Dresden, Germany

3 PowerOn Ltd., Auckland 1010, New Zealand

*** Correspondence:**

M. Franke
markus.franke@tu-dresden.de

SI 1 Modeling Appendix

In Voigt's notation with engineering shear description, the elasticity matrix for the local description of the active part (see Figure 4) is

$$\underline{\underline{E_a}} = \begin{pmatrix} -\frac{E_a}{\nu^2-1} & -\frac{E_a\,\nu}{\nu^2-1} & 0 \\ -\frac{E_a\,\nu}{\nu^2-1} & -\frac{E_a}{\nu^2-1} & 0 \\ 0 & 0 & \frac{E_a}{2\,\nu+2} \end{pmatrix}$$

with E_a, E_b and ν being the elastic modulus of active and passive layers and the Poisson's ratio. Please note that for the passive middle layer, the influence of the casing is included in E_b. With the layering (with $t_a,\ t_b$ being the thickness of the active and passive layer) according to Figure 4, this leads to the ABD-Matrix

$$\underline{\underline{M}} = \begin{pmatrix} -\frac{2\,E_a\,t_a}{\nu^2-1}-\frac{E_b\,t_b}{\nu^2-1} & -\frac{2\,E_a\,\nu\,t_a}{\nu^2-1}-\frac{E_b\,\nu\,t_b}{\nu^2-1} & 0 & 0 & 0 & 0 \\ -\frac{2\,E_a\,\nu\,t_a}{\nu^2-1}-\frac{E_b\,\nu\,t_b}{\nu^2-1} & -\frac{2\,E_a\,t_a}{\nu^2-1}-\frac{E_b\,t_b}{\nu^2-1} & 0 & 0 & 0 & 0 \\ 0 & 0 & \frac{2\,E_a\,t_a}{2\,\nu+2}+\frac{E_b\,t_b}{2\,\nu+2} & 0 & 0 & 0 \\ 0 & 0 & 0 & -\frac{E_b\,t_b^3}{12\,(\nu^2-1)}-\frac{2\,E_a\left(\frac{t_a^3}{12}+t_a\left(\frac{t_a}{2}+t_b\right)^2\right)}{\nu^2-1} & -\frac{2\,E_a\,\nu\left(\frac{t_a^3}{12}+t_a\left(\frac{t_a}{2}+t_b\right)^2\right)}{\nu^2-1}-\frac{E_b\,\nu\,t_b^3}{12\,(\nu^2-1)} & 0 \\ 0 & 0 & 0 & -\frac{2\,E_a\,\nu\left(\frac{t_a^3}{12}+t_a\left(\frac{t_a}{2}+t_b\right)^2\right)}{\nu^2-1}-\frac{E_b\,\nu\,t_b^3}{12\,(\nu^2-1)} & -\frac{E_b\,t_b^3}{12\,(\nu^2-1)}-\frac{2\,E_a\left(\frac{t_a^3}{12}+t_a\left(\frac{t_a}{2}+t_b\right)^2\right)}{\nu^2-1} & 0 \\ 0 & 0 & 0 & 0 & 0 & \frac{E_b\,t_b^3}{12\,(2\,\nu+2)}+\frac{2\,E_a\left(\frac{t_a^3}{12}+t_a\left(\frac{t_a}{2}+t_b\right)^2\right)}{2\,\nu+2} \end{pmatrix}$$

which is symmetrical due to the symmetric setup. Please note that the stiffening effect of the backbone structures can be included in this formulation by applying anisotropic material behavior. Since the current work focusses on the displacement of the end of the structure – and therefore the bending around the y-axis – this effect is not taken into account.

The temperature term that is used to model the actuation with actuation strain $\varepsilon_1^{\text{act}} = \Theta_1\overline{\alpha}_1$ and $\varepsilon_3^{\text{act}} = \Theta_3\overline{\alpha}_3$ for the respective DEA-layer influence is

$$\begin{pmatrix} n_\alpha \\ m_\alpha \end{pmatrix} = \begin{pmatrix} -\Theta_1\left(\frac{E_a t_a \overline{\alpha_1}}{\nu^2-1}+\frac{E_a \nu t_a \overline{\alpha_1}}{\nu^2-1}\right)-\Theta_3\left(\frac{E_a t_a \overline{\alpha_3}}{\nu^2-1}+\frac{E_a \nu t_a \overline{\alpha_3}}{\nu^2-1}\right) \\ -\Theta_1\left(\frac{E_a t_a \overline{\alpha_1}}{\nu^2-1}+\frac{E_a \nu t_a \overline{\alpha_1}}{\nu^2-1}\right)-\Theta_3\left(\frac{E_a t_a \overline{\alpha_3}}{\nu^2-1}+\frac{E_a \nu t_a \overline{\alpha_3}}{\nu^2-1}\right) \\ 0 \\ \Theta_1\left(\frac{E_a \overline{\alpha_1}\left(\frac{\left(t_a+\frac{t_b}{2}\right)^2}{2}-\frac{t_b^2}{8}\right)}{\nu^2-1}+\frac{E_a \nu \overline{\alpha_1}\left(\frac{\left(t_a+\frac{t_b}{2}\right)^2}{2}-\frac{t_b^2}{8}\right)}{\nu^2-1}\right)-\Theta_3\left(\frac{E_a \overline{\alpha_3}\left(\frac{\left(t_a+\frac{t_b}{2}\right)^2}{2}-\frac{t_b^2}{8}\right)}{\nu^2-1}+\frac{E_a \nu \overline{\alpha_3}\left(\frac{\left(t_a+\frac{t_b}{2}\right)^2}{2}-\frac{t_b^2}{8}\right)}{\nu^2-1}\right) \\ \Theta_1\left(\frac{E_a \overline{\alpha_1}\left(\frac{\left(t_a+\frac{t_b}{2}\right)^2}{2}-\frac{t_b^2}{8}\right)}{\nu^2-1}+\frac{E_a \nu \overline{\alpha_1}\left(\frac{\left(t_a+\frac{t_b}{2}\right)^2}{2}-\frac{t_b^2}{8}\right)}{\nu^2-1}\right)-\Theta_3\left(\frac{E_a \overline{\alpha_3}\left(\frac{\left(t_a+\frac{t_b}{2}\right)^2}{2}-\frac{t_b^2}{8}\right)}{\nu^2-1}+\frac{E_a \nu \overline{\alpha_3}\left(\frac{\left(t_a+\frac{t_b}{2}\right)^2}{2}-\frac{t_b^2}{8}\right)}{\nu^2-1}\right) \\ 0 \end{pmatrix}$$

The solution of the system

$$\underline{0} = \underline{\underline{M}}\begin{pmatrix} \underline{\varepsilon} \\ \underline{\kappa} \end{pmatrix} - \begin{pmatrix} n_\alpha \\ m_\alpha \end{pmatrix}$$

leads to a bending curvature around the y-axis with

$$\kappa_{yy} = \frac{6\left(\alpha_1\Theta_1 E_a t_a^2 - \alpha_3\Theta_3 E_a t_a^2 + \alpha_1\Theta_1 E_a t_a t_b - \alpha_3\Theta_3 E_a t_a t_b\right)}{8\,E_a t_a^3 + 24\,E_a\,t_a^2\,t_p + 24\,E_a\,t_a t_b^2 + E_b t_b^3}$$

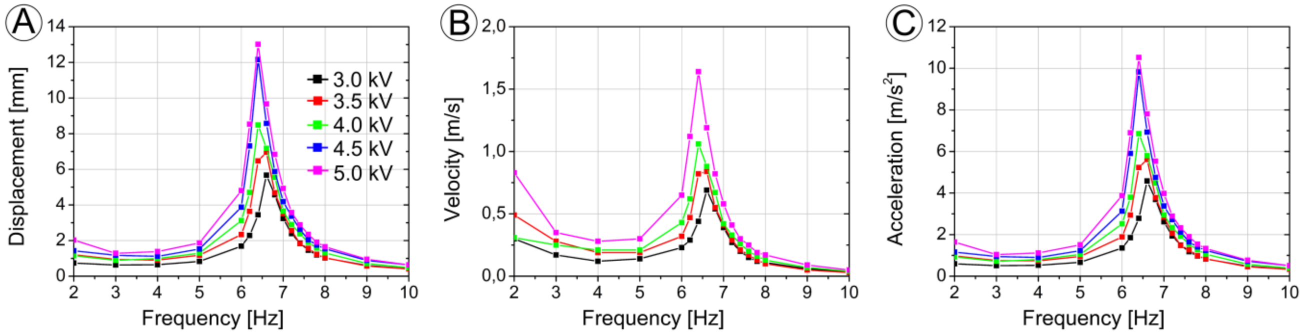

Figure S1: Dynamic investigations of a robotic structure, possessing Moldstar 30 as body material and a λ_{pre} = 1.1 pre-stretched DE membrane, showing the voltage and frequency depending **(A)** dynamic displacement, **(B)** velocity and **(C)** acceleration of the robots tip.

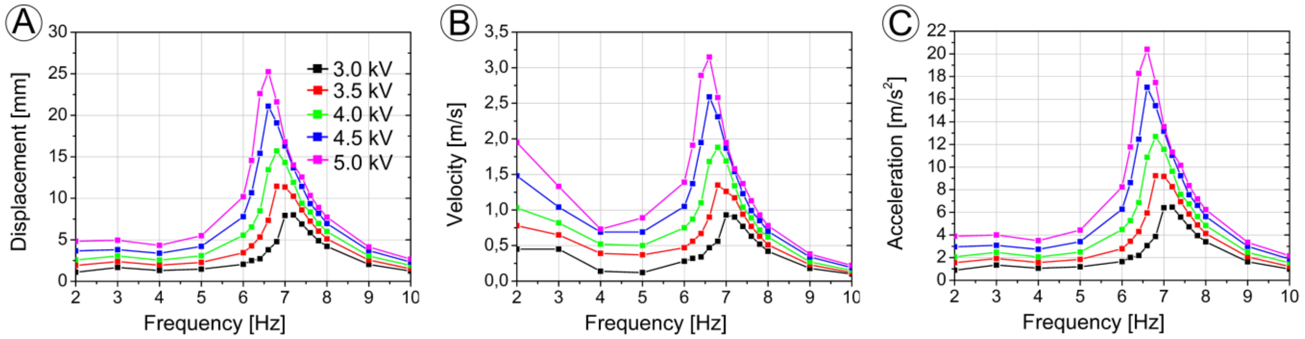

Figure S2: Dynamic investigations of a robotic structure, possessing Moldstar 30 as body material and a λ_{pre} = 1.3 pre-stretched DE membrane, showing the voltage and frequency depending **(A)** dynamic displacement, **(B)** velocity and **(C)** acceleration of the robots tip.

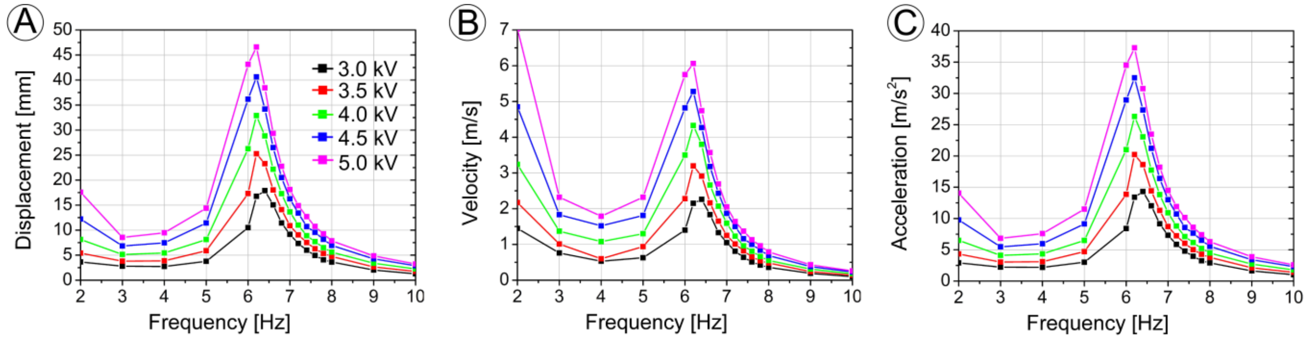

Figure S3: Dynamic investigations of a robotic structure, possessing Moldstar 30 as body material and a λ_{pre} = 1.5 pre-stretched DE membrane, showing the voltage and frequency depending **(A)** dynamic displacement, **(B)** velocity and **(C)** acceleration of the robots tip.

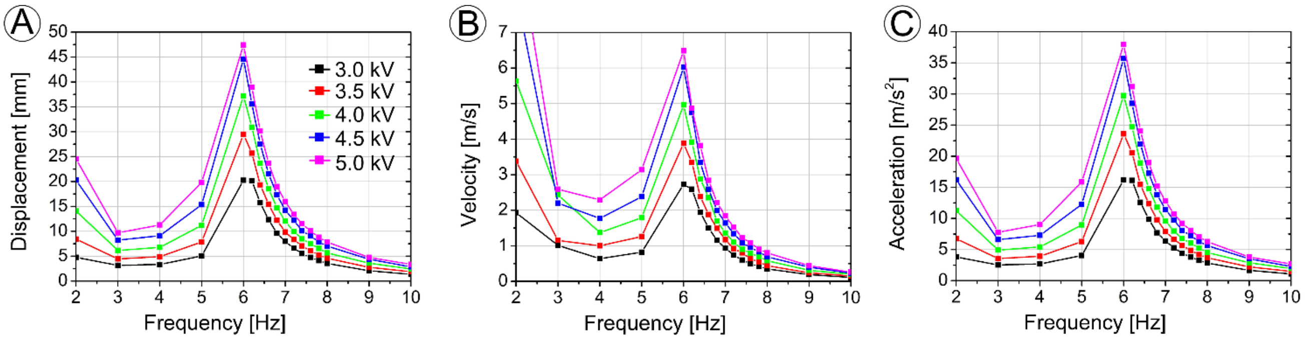

Figure S4: Dynamic investigations of a robotic structure, possessing Moldstar 30 as body material and a λ_{pre} = 1.7 pre-stretched DE membrane, showing the voltage and frequency depending **(A)** dynamic displacement, **(B)** velocity and **(C)** acceleration of the robots tip.

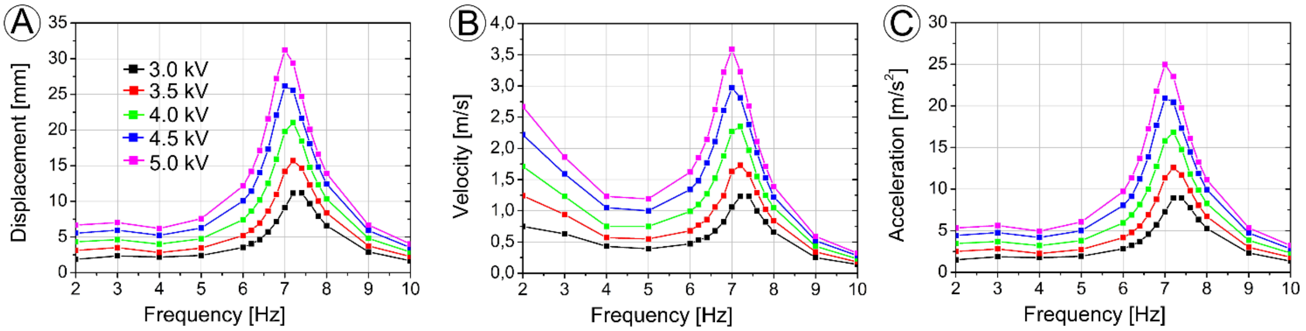

Figure S5: Dynamic investigations of a robotic structure, possessing with Sylgard 184 as body material and a λ_{pre} = 1.5 pre-stretched DE membrane, showing the voltage and frequency depending **(A)** dynamic displacement, **(B)** velocity and **(C)** acceleration of the robots tip.

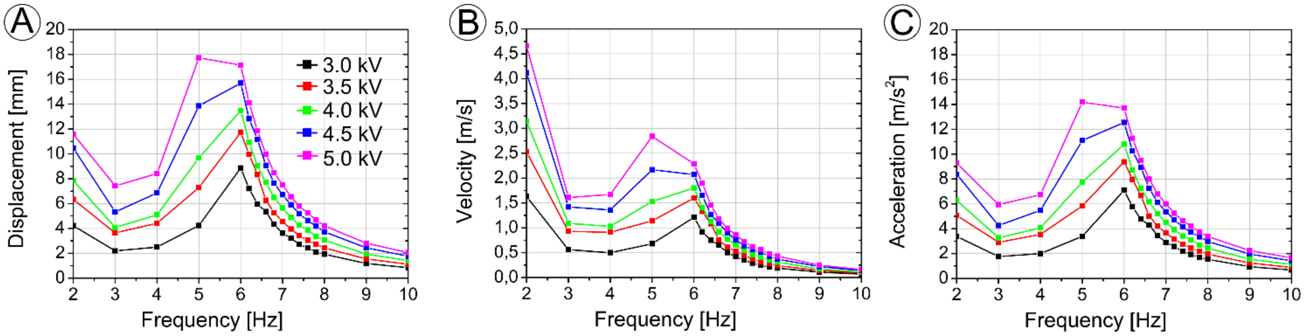

Figure S6: Dynamic investigations of a robotic structure, possessing Ecoflex 00-10 as body material and a λ_{pre} = 1.5 pre-stretched DE membrane, showing the voltage and frequency depending **(A)** dynamic displacement, **(B)** velocity and **(C)** acceleration of the robots tip.

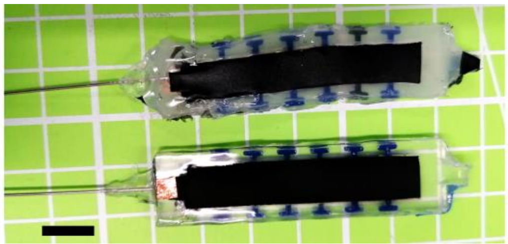

Figure S7: Robots with Ecoflex 00-10 (top) and Sylgard 184 (bottom) silicone bodies showing different DEA membrane and body deformation due to the highly varying Young's moduli. Scale bar is 10 mm.
